# Breast Implants: Biomaterials, Surfaces, Biocompatibility—A Biomedical Engineering Perspective

**DOI:** 10.3390/jcm15114031

**Published:** 2026-05-22

**Authors:** Angelika Auguścik, Julia Lisoń-Kubica, Karolina Wilk, Anna Taratuta, Gabriela Wielgus, Julia Kolasa, Agata Piątek, Inga Szotowska, Magdalena Antonowicz-Hüpsch, Barbara Rynkus

**Affiliations:** 1 Department of Biomaterials and Medical Devices Engineering, Faculty of Biomedical Engineering, Silesian University of Technology, Roosevelta 40 Street, 41-800 Zabrze, Poland; angelika.auguscik@polsl.pl (A.A.); karolina.wilk@polsl.pl (K.W.); anna.taratuta@polsl.pl (A.T.); gabriela.wielgus@polsl.pl (G.W.); julia.kolasa@polsl.pl (J.K.); agata.piatek@polsl.pl (A.P.); barbara.rynkus@polsl.pl (B.R.); 2 SKN SYNERGIA, Department of Biomaterials and Medical Devices Engineering, Faculty of Biomedical Engineering, Silesian University of Technology, Roosevelta 40 Street, 41-800 Zabrze, Poland; is312194@student.polsl.pl

**Keywords:** breast implants, aesthetic surgery, modifications, biomaterials

## Abstract

Breast implants are among the most frequently used long-term implantable medical devices in aesthetic and reconstructive surgery. In addition to correcting anatomical deficits, they have significant psychosocial effects, influencing body image, self-esteem, and quality of life, particularly in patients undergoing postmastectomy reconstruction. This review provides a comprehensive overview of the historical development, biological interactions, material characteristics, and clinical outcomes of breast implants. Early reconstructive attempts using foreign materials and injectable substances were associated with severe complications, underscoring the need for safer technologies. The introduction of silicone gel implants in the 1960s marked a pivotal advancement, followed by the development of saline-filled devices and highly cohesive silicone gels with enhanced mechanical stability. Key surgical considerations, including incision type and implant placement plane (subglandular, submuscular, dual-plane, and subfascial), are discussed in relation to aesthetic outcomes and complication risk. Emphasis is placed on the implant–tissue interface and the foreign body response (FBR), a process involving protein adsorption, immune cell activation, fibrous capsule formation, and potential chronic inflammation. Persistent inflammatory stimulation, often associated with bacterial biofilm formation, contributes to capsular contracture, the most common long-term complication. Additional adverse events include implant rupture, silicone gel bleed, granulomatous reactions, infection, hematoma, implant malposition, and rare but clinically significant conditions such as breast implant-associated anaplastic large cell lymphoma (BIA-ALCL). The review also summarizes implant classification according to construction, filling material, shape, and surface topography, highlighting the influence of surface characteristics on host response and clinical outcomes. Advances in biomaterials, cohesive gel formulations, and surface engineering aim to enhance biocompatibility and long-term safety, supported by standardized mechanical and biological testing protocols.

## 1. Introduction

Breast implants used in both aesthetic and reconstructive surgery are among the most used implantable medical devices. Their use is not limited to the correction of anatomical defects but also involves important psychosocial aspects. Numerous studies indicate that breast appearance plays an important role in shaping body image, sense of femininity, and self-esteem. In patients who have undergone mastectomy, breast reconstruction may contribute to improved quality of life, reduction in depressive and anxiety symptoms, and facilitation of psychological adaptation following cancer [1]. In aesthetic surgery, motivations for implant placement often stem from the desire to improve appearance, conform to cultural standards of beauty, and enhance subjective perceptions of attractiveness; however, the relationship between aesthetic procedures and mental health remains complex and multifaceted [2,3].

The history of breast implants encompasses the development of surgical techniques, material technologies, and evolving medical and aesthetic indications. Early attempts at breast reconstruction and augmentation, undertaken from the late nineteenth to the early twentieth century, relied on materials and techniques that did not meet modern standards of safety and efficacy, giving them an experimental character. A breakthrough occurred in the second half of the twentieth century with the introduction of silicone implants, marking the beginning of modern breast surgery. In the twenty-first century, breast implants have become advanced medical devices designed for both aesthetic and reconstructive applications, incorporating increasingly precise biomedical technologies [4,5]. The earliest attempts at breast reconstruction did not involve the use of implants as understood in modern implantology. Surgical interventions were aimed at restoring tissue deficits following mastectomy and improving the patient’s anatomical condition, rather than aesthetic augmentation [6]. In 1895, Vincenz Czerny performed a procedure considered the first documented breast reconstruction, involving the transplantation of a lipoma from the lumbar region of the back to the site of the defect following removal of the mammary gland [7]. The method used, based on autologous tissue, had limited applicability; however, it demonstrated the feasibility of surgical breast reconstruction.

In subsequent years, attempts were made to use various foreign materials, such as ivory, glass balls, ground rubber, bovine cartilage, and sponges, which were implanted to provide breast volume [7]. Concurrently, at the turn of the nineteenth and twentieth centuries, reports emerged on breast contouring using injectable substances. In 1899, Gersuny described the use of paraffin; however, it was not until the early twentieth century, including in 1911, that more widely cited clinical reports documenting the associated complications were published. Reported complications included the formation of paraffinomas, breast deformities, material migration, ulceration, and tissue necrosis, representing some of the earliest scientific descriptions of adverse effects following filler injections.

In the 1940s, attempts were made to increase breast volume using injections of various liquid substances, including paraffin and petroleum jelly. In subsequent decades, injections of liquid silicones were also used, including both industrial- and medical-grade materials. These procedures were often performed outside the scope of standard medical practice, frequently without appropriately qualified personnel and without control over the volume of material administered. Clinical reports documented numerous local and systemic complications, including chronic inflammatory reactions, infections, ulceration, tissue necrosis, and other systemic disorders [7]. In 1961, Uchida described the injection of liquid silicone (polydimethylsiloxane) into the breast for augmentation, which was associated with frequent complications, including chronic inflammation, infections, granuloma formation, and tissue necrosis; the technique was eventually abandoned due to unacceptable clinical risk [8]. In 1954, Longacre described surgical breast augmentation using a dermal–fat flap. In subsequent years, other autologous tissues, such as adipose tissue and the omentum, were also investigated in breast reconstruction [8,9]. In 1962, Cronin and Gerow introduced the first breast implant consisting of a silicone shell filled with silicone gel, and in 1964, Laboratoires Arion developed a saline-filled implant, providing an alternative to gel-filled devices [10,11].

In the late 1980s and early 1990s, concerns emerged regarding the safety of silicone gel-filled implants. In 1992, the U.S. Food and Drug Administration (FDA) imposed a moratorium on the use of silicone implants for cosmetic purposes in the United States due to insufficient data on their safety and efficacy; however, these implants could still be used for breast reconstruction and in clinical trials [12,13]. In response to these controversies and the moratorium, interest in saline-filled implants increased in the 1990s, as they were approved for use without restrictions. Since the late twentieth century, efforts have continued to further improve implant materials and design, including the development of more cohesive silicone gels, improved shells, and a variety of shapes and surface textures, with the aim of reducing complication rates and improving aesthetic outcomes [14].

Data from the ISAPS Global Survey, the most comprehensive annual survey of aesthetic procedures conducted by the International Society of Aesthetic Plastic Surgery (ISAPS), indicate that surgical breast augmentation remains one of the most performed procedures worldwide. In 2024, breast augmentation ranked third among the most performed surgical procedures, with more than 1.6 million procedures performed globally, surpassed only by liposuction and eyelid surgery (blepharoplasty) [15]. The 2023 ISAPS report also ranked breast augmentation among the most popular surgical procedures, with nearly 1.9 million procedures performed globally, making it one of the most dominant procedures in aesthetic surgical practice worldwide.

**Figure 1:**
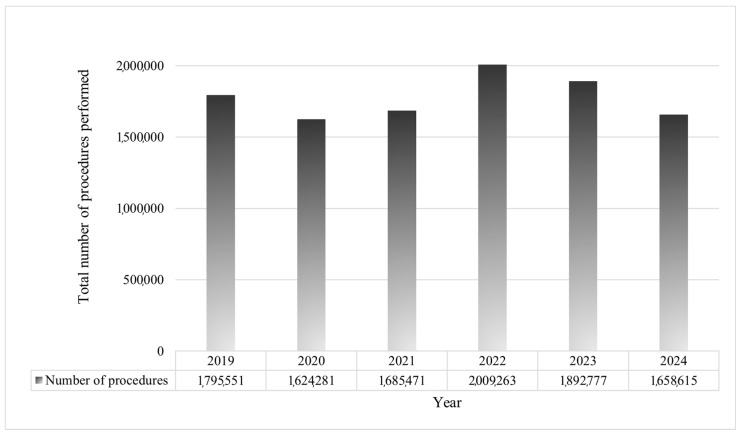
Global statistics on breast reconstruction procedures over the years 2019–2024 according to the ISAPS Global Survey show number of procedures performed in these years.

Global statistics on breast reconstruction and augmentation procedures are presented in Figure 1. Data from the global ISAPS survey indicate that the number of breast implant procedures remains high and has shown an upward trend in recent years, despite a short-term decline of 9.5% during the COVID-19 pandemic in 2020 compared to 2019 [15]. The rapid rebound in subsequent years reflects both the increasing global availability of aesthetic surgery and documented advances in implant technology. Specifically, the introduction of highly cohesive, form-stable silicone gels has been shown to provide a comprehensive safety and effectiveness profile over the long term. For instance, a 10-year prospective core study of cohesive silicone gel implants demonstrated an overall patient rupture rate of 8.6% and a severe capsular contracture (Baker Grade III/IV) rate of 13.5% across all cohorts, with textured devices showing an even lower contracture rate of 9.0% [16,17]. These measurable improvements in safety and aesthetic outcomes directly correlate with the sustained global demand for these procedures.

## 2. Breast Implant Placement

Breast implant placement is a surgical procedure aimed at breast augmentation, reconstruction, or shape enhancement. Its proper performance requires careful preoperative planning, including the selection of the incision site and the implantation plane, which are critical for aesthetic outcomes, safety, and durability of the achieved result.

**Figure 2:**
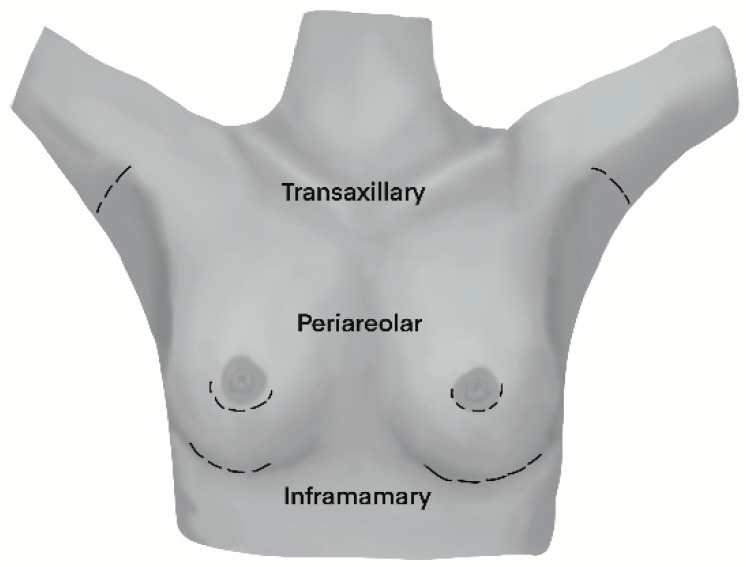
Incision sites, schematic anterior view of the female torso with marked locations of common breast implant incisions (transaxillary, periareolar, inframammary), illustrating typical surgical access routes used in augmentation procedures.

The female breast consists of adipose, connective, and glandular tissue. The mammary gland is composed of 15–20 lobes, from which the milk ducts arise, carrying milk to the lactiferous sinuses, which then open at the nipple. Adipose and connective tissue surround and protect the ducts while also determining the overall shape of the breast. The ducts are thin tubular structures that transport milk to the nipple. Neoplastic changes in the breast may arise in both the ducts and the lobes. A key aspect in this context is also the complex three-dimensional morphology of the breast [18,19]. Before breast implant placement, an incision must be made in the inframammary region, around the areola (periareolar), or in the axillary region (transaxillary) [20]. A less commonly used technique is a transumbilical incision, which allows implant insertion without leaving visible scars on the breasts (Figure 2). This approach is not suitable for silicone implants due to the high risk of shell damage during manual insertion through the narrow umbilical incision [21,22]. Among the available techniques, inframammary incisions are most commonly used, as they provide optimal access to the implantation site.

**Figure 3:**
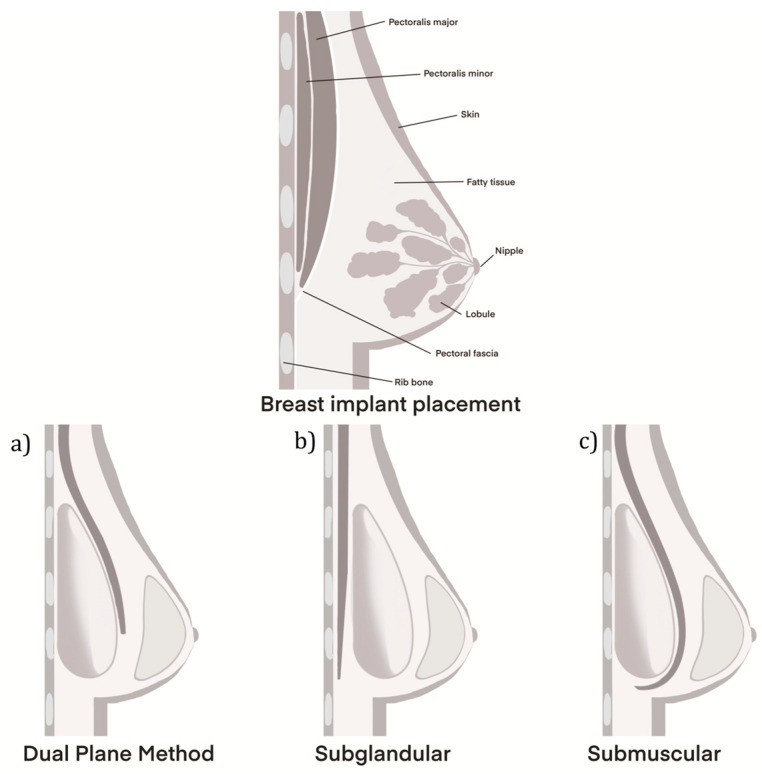
Breast anatomy and method of breast implant placement: (**a**) dual-plane, (**b**) subglandular placement, (**c**) submuscular placement show internal structure of the breast.

The location of the implant pocket depends on the selected implantation plane, as it determines the final aesthetic outcome, risk of complications, and long-term stability. In this context, several options are distinguished, including submuscular implantation—either partially or completely beneath the pectoralis major muscle, and subglandular implantation—above the muscle, beneath the glandular tissue (Figure 3). In clinical practice, subglandular implantation is often used, in which the implant is placed between the mammary gland and the pectoralis major muscle, allowing for the most natural aesthetic outcome. However, in patients with thin soft-tissue coverage, implant visibility and palpability may be increased [23]. An alternative approach is submuscular implantation, which involves placing the implant beneath the pectoralis major muscle and is associated with a lower risk of capsular contracture [24,25]. A modification of submuscular implantation is the dual-plane technique, which involves partial release of the pectoralis major muscle, allowing the upper part of the implant to be covered by muscle while the lower part lies in a subglandular position [26]. Subfascial implantation is a technique in which the breast implant is placed beneath the fascia of the pectoralis major muscle. This technique may be a favorable option in patients with limited soft-tissue coverage in the upper pole of the breast. Proponents of this approach point to its potential benefits, including protection against capsular contracture comparable to submuscular implantation; however, the available scientific evidence does not provide unequivocal support for this claim [27,28,29].

In prepectoral (subcutaneous) placement, the implant is positioned above the pectoralis major muscle, without the need for muscle dissection and is typically placed directly beneath the mastectomy skin flap. To provide adequate support and improve soft-tissue coverage, the implant is often wrapped in or covered with acellular dermal matrices (ADM) or synthetic meshes. The use of ADM in breast reconstruction was first introduced in 2006 by Saltzberg, who described its successful applications [30,31]. ADM serves as a scaffold for tissue and cellular ingrowth while minimizing concomitant fibrosis and inflammation. The advantages of this method include shorter operation time and faster recovery, primarily because the pectoralis major muscle remains intact. However, it requires adequate vascularization of the skin flap and sufficient subcutaneous tissue thickness. In cases of thin skin flaps, the risk of implant palpability increases [32].

Nevertheless, selection of the breast implant placement plane is critical for achieving the desired outcome and minimizing the risk of complications. Available systematic reviews indicate that no single technique represents a universal solution, and each is associated with specific benefits and limitations. The decision is typically determined by the surgeon’s preferences and experience, as well as breast anatomy [33].

## 3. Reactions at the Implant–Tissue Interface

Following breast implant placement, the body recognizes the implant as a foreign body. In response, wound healing processes are initiated, along with the foreign body response (FBR). These processes lead to tissue repair and structural remodeling. However, the persistent presence of the implant sustains immune system activation and promotes the maintenance of an inflammatory response of varying intensity. A natural consequence is the formation of a fibrous capsule, i.e., a thin layer of scar tissue surrounding the implant that mechanically isolates it from the surrounding tissues [34]. However, studies indicate that within the capsule and in the immediate vicinity of the implant surface, an inflammatory state of varying intensity may persist, promoting adverse tissue remodeling and potentially contributing to later clinical complications. These reactions may lead, among others, to thickening and hardening of the fibrous capsule, pain, and breast deformity, and in more severe cases increase the likelihood of surgical intervention. Excessive capsular scarring leads to progressive stiffening of the capsule, which may mechanically compress and damage the implant. As a result, the breast may become firm, painful, and deformed, and in some cases implant displacement may occur [35]. An important group of phenomena at the implant–tissue interface involves microbiological colonization of the implant surface and the formation of an organized bacterial biofilm. Such biofilms do not necessarily produce the typical signs of acute infection, but can sustain long-term, low-grade inflammatory stimulation within the capsule and influence its remodeling [36]. A growing body of evidence, including multicenter sonication-based studies, has demonstrated that positive bacterial cultures from explanted devices (most commonly *Staphylococcus epidermidis*, *Cutibacterium acnes*, and *Coagulase-negative staphylococci*) correlate with the severity of capsular contracture [37]. A meta-analysis, regarded as the highest level of evidence, indicates that despite its widespread use, antibiotic prophylaxis (both local and systemic) does not demonstrate statistically significant effectiveness in preventing capsular contracture [38]. The etiology of capsular contracture is considered multifactorial, and a direct causal relationship between biofilm formation and contracture has not been definitively established [39]. Another mechanism at the implant–tissue interface involves contact of the capsule with particles of the material or small amounts of implant contents, which may be present even in the absence of visible rupture. The immune system then responds by attempting to eliminate the foreign material, which may lead to the development of foci of chronic inflammation within the capsule (granulomatous reaction). Clinically, this may present as a lesion within the capsule on imaging studies or as discomfort; it is also described in association with capsular thickening [40]. The most common reactions at the implant–tissue interface are summarized in Table 1.

**Figure 4:**
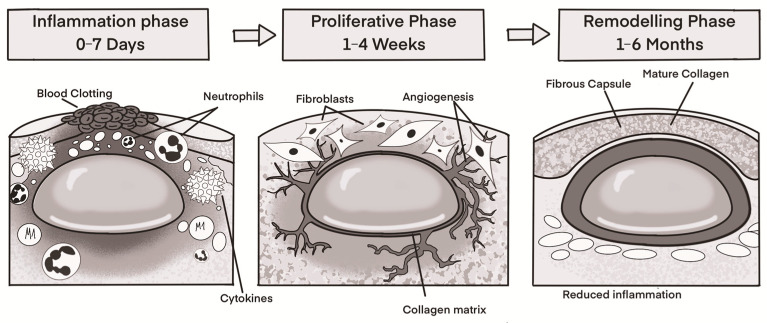
Schematic representation of the wound healing process at the implant–tissue interface, including the hemostasis, inflammatory, proliferative, and remodeling phases, along with the major cellular and biological events involved in tissue regeneration and implant integration, M1 means—**M1 inflammation** refers to the activation of **M1 macrophages**—white blood cells that drive the body’s primary pro-inflammatory immune response.

The observed clinical findings result from the activation of specific biological mechanisms that determine the course of the healing process at the implant–tissue interface. Tissue injury initiates a sequence of events comprising the hemostasis, inflammatory, proliferative, and remodeling phases (Figure 4) [34].

Table 2 presents the successive stages of the tissue healing process along with the underlying biological mechanisms.

The foreign body response (FBR) is an immune response to the presence of an implant, leading to formation of a fibrous capsule surrounding the implant. This phenomenon is similar to physiological wound healing; however, it results in complete separation of the breast implant from surrounding tissues [45]. The stages of the foreign body response (FBR) and the underlying mechanisms are summarized in Table 3.

From a clinical perspective, the foreign body response (FBR) may lead to persistent inflammation through continuous cytokine release and fibroblast activation, ultimately resulting in capsular contracture. Macrophage activation promotes the release of profibrotic mediators, which causes fibroblast differentiation into myofibroblasts and excessive collagen deposition [34,45]. Unlike wound healing, which resolves after scar tissue formation, the foreign body response (FBR) promotes prolonged ECM remodeling and capsular contracture formation, which may negatively affect breast implant functionality [34,45].

## 4. Complications

Complications following breast implant placement include both early events related to the healing process (implant–tissue interface reactions) and later events that may be associated with the mechanical durability of the implants or their wear. Some complications are mild; however, others (such as mechanical implant damage) may be very dangerous and increase the likelihood of another surgical intervention. The most common complications are summarized in Table 4. Complications most often arise from two causes: impaired healing and changes in tissues surrounding the implant, and damage to or displacement of the implant itself. Complications should not be overlooked and should be consulted with a physician in each case.

## 5. General Classification of Breast Implants

In the classical approach, five generations of breast implants are distinguished, reflecting successive stages in implant technology evolution. In the latest generations of silicone gel-filled implants, a cohesive, highly viscous silicone gel is used, preventing, in contrast to earlier models, shell collapse even in the event of rupture. In the third and fourth generations, implants with textured and smooth surfaces were introduced, which contributed to a reduced incidence of capsular contracture. The latest generations are mechanically more stable and are associated with a lower risk of silicone migration [52].

**Figure 5:**
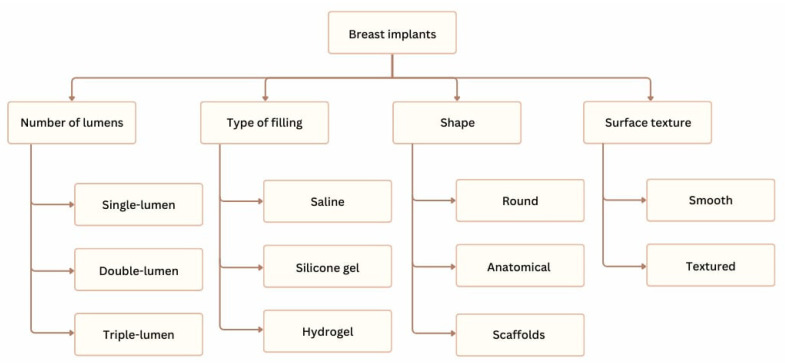
Classification of breast implants is divided with number of lumens, type of filling, shape and surface texture.

Breast implants are classified according to their design, type of filler material, and shape (Figure 5), reflecting the range of available options and allowing selection of an implant tailored to individual patient needs.

### 5.1. Classification of Breast Implants by Design

The most commonly used devices are single-chamber implants, consisting of a single chamber filled with silicone gel and surrounded by a multilayer shell [52,53,54]. Earlier designs used lower-viscosity gel, which could easily migrate into surrounding tissues in the event of rupture. Contemporary implants contain highly cohesive gel that maintains its shape even after shell damage [53].

Double-chamber implants consist of an outer silicone chamber and an inner chamber filled with saline, the volume of which can be adjusted via a subcutaneous filling port [52,53]. The best-known example is the Becker implant, used primarily in staged breast reconstruction. This design allows gradual volume expansion, greater flexibility in reconstruction planning, and additional safety in the event of failure of one of the chambers [54,55].

Trilucent implants were filled with substances of low radiographic density (e.g., soybean oil) to enhance mammographic transparency [53]. Despite initial enthusiasm, these implants were withdrawn from the market due to chemical instability of the filler material, potential biological reactivity, and the occurrence of artifacts in MRI studies.

### 5.2. Classification of Breast Implants by Filler Material and Shape

Depending on the type of filling material, implants can be classified as saline-filled or silicone gel-filled. Saline-filled implants, described by Spear and Jespersen [56], have a flexible silicone shell that is filled with saline after placement in the implant pocket. Advantages of this approach include the ability to adjust implant volume intraoperatively and easy detection of deflation. Disadvantages include a greater tendency for shell wrinkling and a less natural feel. Silicone gel-filled implants (with varying degrees of gel cohesiveness) provide a more natural consistency and reduced visibility of shell folds [54,56].

**Figure 6:**
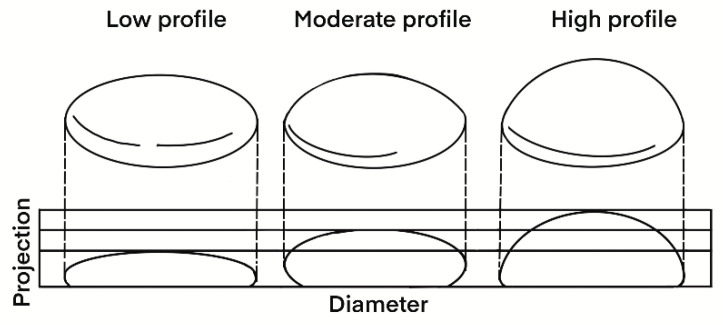
Comparison of the shapes of low-, medium-, and high-profile implants. The diameter defines a fixed anatomical width, while the projection distinguishes implants based on their height. This configuration allows the surgeon to perform precise augmentation in patients with a narrow chest who require significant shape correction.

Differences in implant design are also reflected in their shape, which affects aesthetic outcomes. In this category, implants can be broadly divided into round and anatomical types (Figure 6). The primary parameters defining an implant and the outcomes achievable with its use include base diameter, volume, projection, and profile [57].

Implants are available with:

- Low profiles, characterized by a wide base and a slightly flattened breast shape, suitable for patients with a broad chest;
- Medium profiles, providing the most natural appearance;
- High profiles, after placement resulting in a more spherical breast shape with greater anterior projection, recommended for patients with a narrow chest [58].

**Figure 7:**
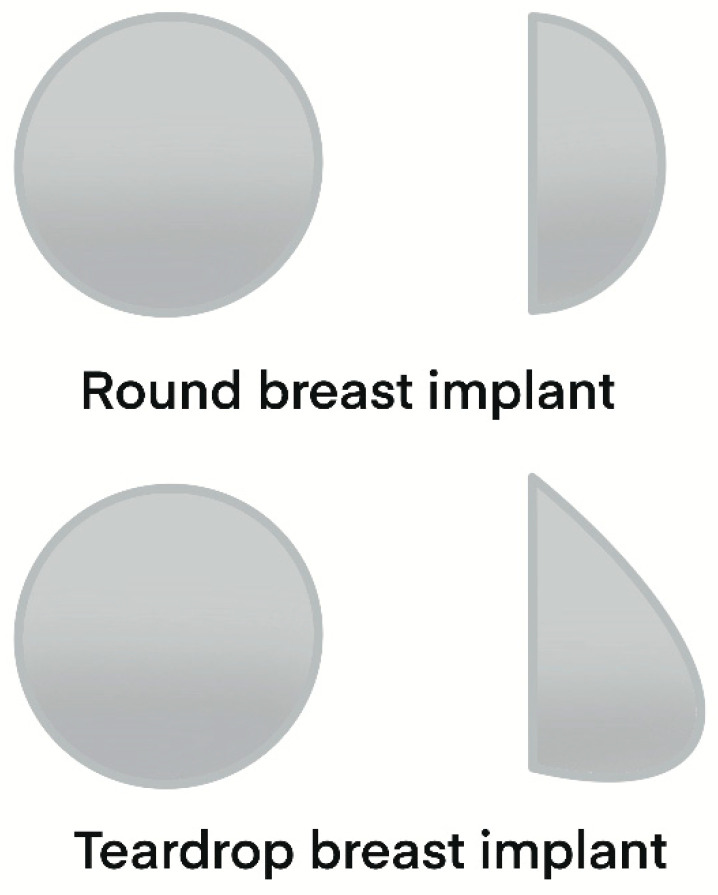
Shapes of breast implants. The illustration compares two types of breast implants shown in front and side views: a round implant and an anatomical teardrop-shaped implant. The round implant has a symmetrical, dome-like profile, while the teardrop implant features an asymmetrical shape with greater fullness in the lower portion, mimicking the natural contour of the breast.

In the clinical literature, profile is defined as the ratio of base diameter to implant volume (Figure 7) [59].

A study by Bletsis et al. showed that anatomical implants are perceived as more natural by both surgeons and non-specialists [60]. Round implants provide greater fullness of the upper breast pole, whereas anatomical implants more closely replicate the natural breast contour. Differences in height, width, and projection further influence implant selection based on patient anatomy and aesthetic expectations.

### 5.3. Classification of Breast Implants by Size

Breast implants can be classified according to their volume, which is typically expressed in cubic centimeters (cc) or milliliters (mL). Implant size is a key parameter influencing the final breast volume and overall aesthetic outcome following breast augmentation or reconstruction procedures. The selection of implant size should take into account patient-specific anatomical characteristics, including chest wall dimensions, existing breast tissue, skin elasticity, and individual aesthetic expectations. From a clinical perspective, breast implants may be categorized into several size groups (Table 5) [61].

In clinical practice, the appropriate implant size is determined through a comprehensive evaluation of anatomical parameters, patient preferences, and surgical considerations, with the aim of achieving optimal aesthetic results while minimizing the risk of postoperative complications.

### 5.4. Breast Implant Texture

Based on surface characteristics, breast implants are divided into two main categories: smooth and textured. Numerous meta-analyses indicate that smooth implants are more prone to capsular contracture, i.e., the most common complication following breast augmentation. Accordingly, study findings suggest that the use of textured implants significantly reduces the incidence of capsular contracture compared with smooth implants [60,62,63]. Texturing plays a key role in shaping the biological response and enabling further chemical functionalization of silicone. Modification of surface topography at the micro- and nanoscale affects cell and microorganism adhesion; however, its biological effect is strongly dependent on the scale and nature of the structure [39,64]. Differences between textured and smooth implants primarily involve the nature of the foreign body response (FBR) and fibrous capsule formation. Textured implants enhance the immune response, including macrophage activation and a tendency for bacterial biofilm formation, leading to an increased risk of infection. In contrast, in smooth implants, collagen fibers are arranged parallel to the implant surface. However, this structural organization may increase the risk of capsular contracture. Textured surfaces were originally developed to disrupt the parallel alignment of collagen fibers at the implant interface and thereby reduce capsular contracture [65].

The comparative evidence is, however, heterogeneous and context-dependent. Meta-analyses in primary cosmetic augmentation have consistently reported lower contracture rates with textured implants. For example, a 2022 meta-analysis including 13 studies demonstrated an odds ratio in favor of textured implants [55]. Other studies indicate that smooth implants are associated with a significantly higher risk of capsular contracture compared to textured implants, and the use of textured implants may reduce this risk [66]. In contrast, recent systematic reviews in the reconstructive setting have not confirmed this advantage. A 2024 meta-analysis found no significant difference between smooth and textured implants in implant-based breast reconstruction [67], while an analysis of direct-to-implant reconstruction (in the context of breast reconstruction after mastectomy) reported significantly lower contracture rates with smooth round implants than with textured anatomical devices [68]. These discrepancies likely reflect differences in patient populations, implant placement plane, use of acellular dermal matrices, and follow-up duration, and are consistent with the multifactorial etiology of capsular contracture emphasized in recent systematic reviews [57]. Mechanistically, textured shells also exhibit a greater tendency for bacterial biofilm formation, which may partly offset their benefit on contracture rates [58].

Importantly, the potential benefit of textured implants with respect to capsular contracture must be weighed against their association with breast implant-associated anaplastic large cell lymphoma (BIA-ALCL), which occurs almost exclusively in patients exposed to textured devices [69]. As a result, clinical practice (particularly in North America) has shifted substantially toward smooth-surface implants [70], and the contemporary choice between smooth and textured devices requires individualized weighing of the lower contracture rate reported for some textured designs against the rare but serious risk of BIA-ALCL.

### 5.5. Autologous Breast Reconstruction

In addition to implant-based techniques, autologous musculocutaneous flaps are also used, such as the TRAM flap and the latissimus dorsi muscle flap. They may be used alone or in combination with an implant, providing additional volume and a more natural breast contour [52]. Currently, however, the gold standard in autologous breast reconstruction is the skin and fat flap based on perforators of the deep inferior epigastric artery (deep inferior epigastric perforator, DIEP), introduced into breast surgery by Robert Allen in 1994, building on earlier work by Koshima and Soeda on perforator flaps that spare the rectus abdominis muscle [6,7]. In contrast to the classic TRAM flap, the DIEP flap enables transfer of skin and adipose tissue from the abdominal wall while preserving the continuity of the rectus abdominis muscle, which significantly reduces donor-site morbidity, including abdominal wall weakness and the risk of postoperative hernias [6]. Allen and Treece, in their initial series of 15 reconstructions, demonstrated that a flap based on one to three perforators of the deep inferior epigastric vessels provides the same aesthetic benefits as the TRAM flap, while causing significantly less damage to the abdominal wall. Subsequent studies by Blondeel in a cohort of 100 DIEP reconstructions confirmed the reproducibility and safety of this technique [6]. From the perspective of patient-reported outcomes, data from a systematic review by Roy et al. indicate that DIEP is the most commonly used autologous method in the contemporary literature, and patients undergoing DIEP reconstruction report the highest levels of satisfaction with breast appearance among the compared methods, as well as a better body image than patients undergoing implant-based reconstruction [3].

A review of manufacturers’ catalogs confirms efforts to eliminate implant limitations and enhance safety, biocompatibility, and aesthetic outcomes intended to replicate the natural appearance of the breast (Table 6). Observations of outcomes and complications associated with breast implant use, together with rapid advances in manufacturing technologies, have led to the development of numerous implant models.

In recent years, research in breast reconstruction has extended beyond traditional silicone-based implants toward tissue engineering and biofabrication strategies, offering fundamentally new clinical possibilities. Rather than passive volume replacement, these approaches use patient-specific, three-dimensional printed scaffolds and biofabrication techniques to create constructs tailored to the individual patient and can guide and support regeneration of native adipose tissue and vascular networks, potentially leading to more durable, anatomically accurate, and functional outcomes than conventional implants [75]. Three-dimensional printing combined with tissue engineering aims to overcome limitations of existing methods, such as volume loss after fat grafting and the foreign body response. This is possible by enabling the creation of constructs tailored to individual anatomy and the biological environment that may facilitate single-stage procedures with excellent aesthetic and sensory reconstruction [76].

Modern scaffolds are designed as highly porous, open, three-dimensional structures with controlled architecture (e.g., multilayer, dome-shaped designs with pillars and an open base) that provide shape stability, allow direct contact with the host vascular bed, and promote rapid vascularization and survival of transplanted adipose tissue or cells [76,77]. This approach enables personalization of geometry to patient anatomy, improved biological integration, and more natural mechanical properties of the reconstructed breast, representing an important step toward bioactive, regenerative implants instead of passive prostheses.

Evidence from preclinical and early clinical studies suggests that such constructs may enhance adipose tissue regeneration, improve contour and volume retention, and support vascularization, representing a significant shift in reconstructive strategies toward regenerative medicine principles and patient-specific care [78,79].

Modern clinical practice relies on pre-pectoral reconstruction, acellular dermal matrices, synthetic meshes, and advanced implant technologies [80]. Acellular dermal matrices are composed of collagen-rich extracellular matrix decellularized from human or animal tissues. Biological meshes promote collagen remodeling and this property of tissue formation results in effective implant coverage. In contrast, synthetic meshes, such as titanium-coated polypropylene meshes, create a structural scaffold that supports fibrous tissue ingrowth. In a clinical study including 42 patients, histological evaluation of titanium-coated polypropylene meshes demonstrated complete mesh with newly vascularized fibroblastic tissue [81,82,83]. Modern breast reconstruction, particularly in the context of anterior breast techniques, is one of the fastest-growing areas of oncoplastic surgery. Recent developments reflect a shift toward less invasive procedures combined with improved aesthetic outcomes. A major milestone in this evolution was the introduction of acellular dermal matrices (ADM), which enable more stable implant support, better control of implant positioning, and more natural reconstruction results. Despite their widespread use and structural advantages, current evidence does not unequivocally confirm the superiority of ADM in reducing postoperative complications. Systematic reviews and meta-analyses indicate that anterior breast reconstruction performed with and without ADM is associated with comparable complication rates. Although ADM may offer aesthetic and structural benefits in selected patients, its use increases treatment costs and may be associated with additional risks [JLK1]. The use of ADM particularly in subpectoral and direct-to-implant (DTI) breast reconstruction is becoming increasingly common. However, indications for its application and its safety profile are not fully established. Patient qualification depends on factors such as adequate subcutaneous tissue thickness and the absence of prior radiation therapy. Increased complication risk is observed in patients who smoke, have undergone radiotherapy, had large breasts, or require axillary lymphadenectomy. In contrast, factors such as diabetes, BMI, implant size, and age show no clear or consistent correlation with complication rates. Overall, the complication profile of various animal-derived ADMs remains comparable, and current eligibility criteria are consistent with existing recommendations [84]. Further comparative analyses confirm that the use of ADM does not significantly influence the incidence of complications such as seroma, infection, capsular contracture, or tissue wrinkling. Moreover, in patients with well-vascularized post-mastectomy flaps, reconstruction without ADM may be equally safe and effective. These findings suggest that routine ADM use is not always necessary and that decisions should be individualized based on patient-specific factors [85]. Capsular contracture remains one of the key complications of implant-based reconstruction, with a multifactorial etiology. The introduction of ADM, including newer products such as FlexHD Pliable PRE, has improved implant support and tissue integration. However, available evidence largely based on retrospective studies conducted in specialized centers limits the generalizability of these findings. Additionally, confounding factors such as bacterial biofilm complicate the interpretation of ADM’s true impact on capsular contracture risk [86]. In the context of different ADM types, including human-derived products such as AlloDerm and DermACELL, current data remain inconclusive. Variations in processing methods may influence sterility and biocompatibility, but their direct impact on clinical outcomes has not been clearly demonstrated. Ongoing systematic reviews and meta-analyses aim to better evaluate complication rates, patient satisfaction, and overall effectiveness, potentially guiding future material selection and improving clinical outcomes [87]. Another aspect of the investigation is the effect of ADM size on complication rates. Available evidence suggests that matrix size itself does not significantly influence the incidence of complications such as seroma or infection. Observed differences are more likely related to the presence of ADM rather than its dimensions, indicating a relatively stable safety profile regardless of size [88]. At the same time, alternative approaches are being developed to address the limitations of ADM, particularly its cost and uncertain impact on complications. These include synthetic, bioresorbable meshes made from polymers such as poly-4-hydroxybutyrate (P4HB), which function as temporary scaffolds that degrade gradually while supporting tissue regeneration and integration. Preliminary clinical data (with follow-up up to 18 months) suggest that P4HB provides effective implant stabilization, is associated with low reoperation rates, and may improve cost-effectiveness while maintaining patient safety [89]. Simultaneously, advancements in implant technology and biomaterials focus on improving biocompatibility, reducing capsular contracture risk, and better adapting reconstruction to individual patient anatomy. Increasing emphasis is also placed on personalized treatment planning that incorporates both clinical factors and aesthetic expectations. Despite these significant technological and clinical advances, there is still no clear consensus on the optimal breast reconstruction strategy. This highlights the ongoing need for high-quality, prospective, and randomized clinical studies to better define indications, compare available materials, and optimize patient outcomes.

## 6. Biomaterials and Their Modifications Used in Breast Implants

The material from which the implant is made also affects surgical outcomes. The key features to create the ideal implant that need be taken into consideration are: long-term biocompatibility, chemical and mechanical stability, structural integrity of the shell, and controlled interactions with the surrounding tissue. Breast implants are classified as long-term implantable medical devices and therefore must not induce cytotoxic, mutagenic, or carcinogenic responses, while minimizing chronic inflammation and fibrotic capsule formation in accordance with established biocompatibility standards (e.g., ISO 10993) [90]. Silicone elastomers and cohesive silicone gels are currently the materials of choice due to their favorable combination of elasticity, fatigue resistance, and chemical inertness, enabling mechanical behavior similar to soft tissue and durability under repetitive physiological loading [91]. In addition, the permeability and rupture resistance of the outer shell are critical to prevent gel bleed and device failure. Increasing evidence also indicates that surface characteristics, including roughness and texturing, significantly influence host response, capsular contracture rates, and long-term clinical outcomes [92]. Consequently, the development of modern breast implants involves not only optimization of bulk material properties but also advanced surface engineering strategies aimed at improving biological integration and reducing complications [64].

Saline-filled implants are introduced into the body initially empty (silicone shell only) and are then filled with saline. This procedure allows for a smaller incision than with silicone gel-filled implants and therefore results in smaller scars. In addition, removal of these implants is also easier. Saline-filled implants are relatively inexpensive; however, they do not provide a natural breast appearance, may fold and wrinkle along the edges, and are firm to the touch [93].

Silicone gel-filled implants are smoother and more natural in appearance. Both visually and to touch, they closely resemble natural breasts. However, their fill volume is not adjustable, as with saline-filled implants, which requires larger incisions during breast implant placement and results in larger scars. The size of postoperative scars is related to the size of the implanted device. Silicone gel-filled implants are more expensive than saline-filled implants [94].

**Figure 8:**
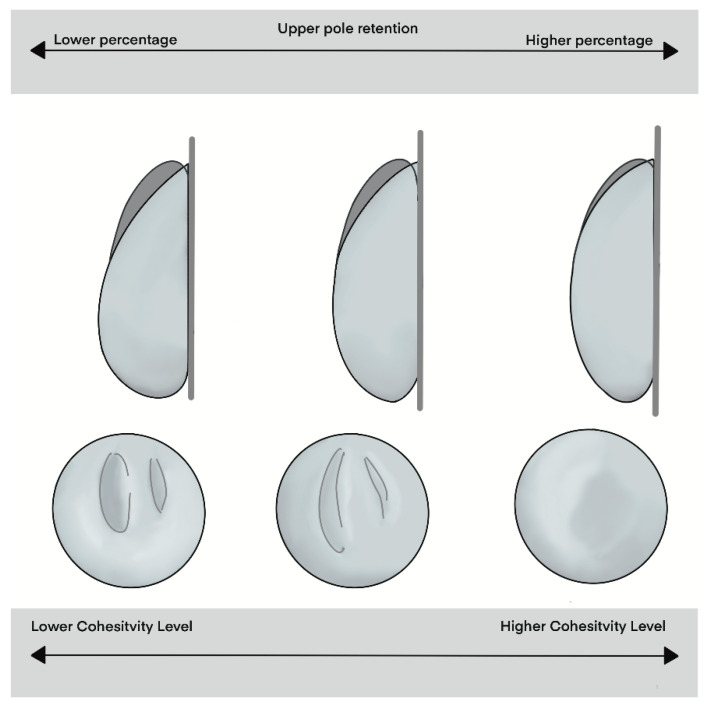
Types of implant cross-linking according to upper pole retention and cohesivity level.

Commonly used implants consist of an elastomer shell made of polydimethylsiloxane (PDMS) [(CH_3_)_2_SiO] filled with silicone gel or sterile saline (0.9% aqueous sodium chloride solution, NaCl) [95]. The difference between the shell and the gel composition lies in the degree of crosslinking between polymer chains; the degree of crosslinking of PDMS in the gel is lower [94]. PDMS is characterized by biocompatibility, flexibility, and thermal and chemical stability. As a result of chemical crosslinking of the silicone gel polymer, a solid form of silicone, known as an elastomer, is formed, with flexible, rubber-like properties. In addition, in modern implant designs, a barrier layer (typically made of fluorosilane or phenylsiloxane) is placed between the layers of the PDMS shell (Figure 8) [94].

PDMS is the primary material used in implant manufacturing; however, various forms of silicone gel crosslinking, depending on the desired outcomes, are offered by manufacturers on the market. Silicone gels can be produced with varying viscosity by progressively increasing the length of polymer chains or the degree of crosslinking.

In both saline-filled and silicone gel-filled implants, the implant shells are manufactured using highly crosslinked PDMS silicone elastomers, forming a rubber-like shell [94].

### 6.1. Surfaces

Many manufacturers modify implant surfaces to achieve the desired roughness. A general classification of implants based on shell surface roughness is presented in Table 7 [93].

Several types of implant shells are distinguished: single or double, smooth or textured, or covered with polyurethane foam. These shells differ from each other depending on the composition and properties of the elastomer used, the number of layers, and the type of barrier layer applied [96]. The shells are designed to reduce the risk of adverse effects associated with implant placement, such as capsular contracture and silicone gel diffusion outside the implant.

### 6.2. Shells Used in Breast Implants

**Figure 9:**
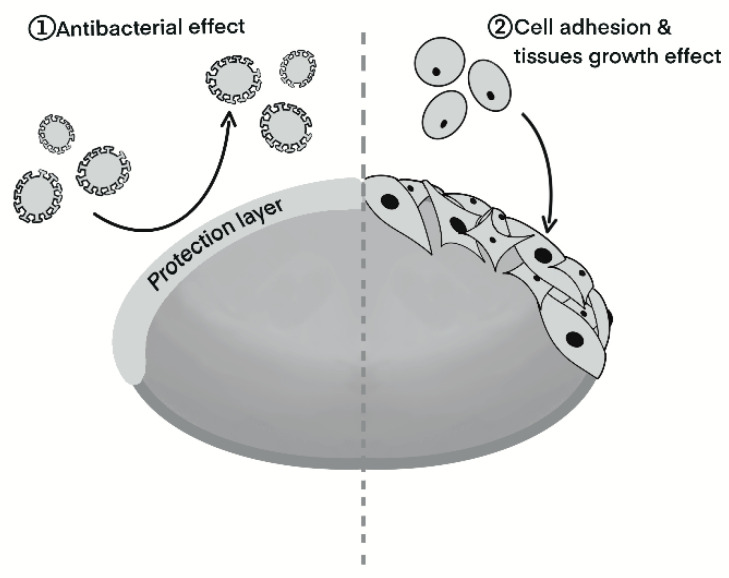
Double functionality for an optimal integration of breast implants inside the body: antibacterial protection and promotion of tissue integration.

Despite the favorable mechanical properties and chemical stability, PDMS is highly hydrophobic and chemically inert, which promotes plasma protein adsorption and secondary bacterial colonization (Table 8). Surface modification using appropriately designed shells is intended to mitigate the most common complications, such as bacterial infections (biofilm formation) and capsular contracture (Figure 9). These effects have been demonstrated primarily in in vitro and preclinical studies, while clinical evidence remains limited and heterogeneous [64].

Due to the chemical inertness and high hydrophobicity of PDMS surfaces, direct functionalization of the material is significantly limited. The silicone surface is dominated by methyl groups, which exhibit minimal chemical reactivity, which limits the stable binding of shells or bioactive molecules. Therefore, the surface activation step is a key element in preparing PDMS for further functionalization. A range of methods for PDMS surface activation has been described in the literature, including plasma treatment [97,98,99], UV/ozone exposure [100], corona discharge [101], as well as chemical activation enabling silanization and covalent attachment of functional layers. Activation introduces polar functional groups, primarily silanol (–Si–OH) groups, increasing surface energy [102,103] and enabling stable anchoring of shells and covalent modifications [64,104].

**Table 8:** Capsular contracture vs. surgical site infections [102].

	Surgical Site Infection	Capsular Contracture
Microorganism	*Staphylococcus aureus*	*Coagulase negative staphylococcus*, *Propionibacterium*, *Bacillus*
Virulence	+	−
Antibiotic prophylaxis	Cephalosporins	Glycopeptides (vancomycin)

The literature describes a wide range of functional coatings applied to silicone surfaces. Antibiotic-based coatings (e.g., cefazolin and gentamicin [104], doxycycline [105], vancomycin [102]) exhibit direct bactericidal activity; however, their long-term effectiveness can be limited by poor stability, drug diffusion, and the risk of resistance development. Coatings based on metal and metal oxide nanoparticles (Ag, ZnO, TiO_2_, CuO [106,107,108,109,110,111,112]) act primarily by inducing oxidative stress or damaging bacterial cell membranes; however, their use requires strict composition control due to potential cytotoxicity. Polymeric and polysaccharide coatings (PEG, hyaluronic acid, chitosan, carboxymethylcellulose [113,114]) are primarily designed as anti-adhesive surfaces, limiting protein adsorption and the initiation of biofilm formation. Bioactive coatings (collagen, NO-releasing coatings, graphene and its derivatives [115,116]) combine antibacterial activity with enhanced biocompatibility and cellular response. Covalent modifications are increasingly recognized for providing greater durability and control of functionality compared with coatings based on physical interactions [64,117,118]. Although numerous surface modifications demonstrate antibacterial and anti-adhesive properties under experimental conditions, their translation into improved long-term clinical outcomes has not yet been consistently confirmed in well-controlled clinical studies.

A critical issue related to the use of coatings is their durability and long-term effects; however, few studies cover such extended timeframes. One of the few sources allowing assessment of these aspects is data from a 30-year follow-up period, indicating that polyurethane-coated breast implants are associated with a significantly lower risk of capsular contracture compared with smooth- and textured-surface implants. The presence of a macroscopic polyurethane coating correlated with the absence of clinical signs of capsular contracture and the maintenance of soft breast consistency. Over time, as the coating degraded, a gradual onset of capsular contracture was observed. However, microscopic presence of polyurethane material in the capsule was still confirmed many years after implantation. These findings are primarily based on long-term observational data and should therefore be interpreted with caution, given the variability in study design and patient populations [119].

In summary, the use of coatings on breast implants represents a promising area for further research, yet their actual impact on long-term clinical outcomes remains to be fully established.

## 7. Studies on Breast Implants—Experimental and Regulatory

Studies on breast implants play a key role in evaluating and ensuring their appropriate physicochemical, mechanical, and material properties. Breast implants are classified as Class III medical devices under Regulation 2017/745, Chapter III, point 5.4—Rule 8: “All implantable and long-term surgically invasive devices are classified as Class IIb, unless they are breast implants or surgical meshes, in which case they are classified as Class III” [120].

It is also important to conduct in vitro and in vivo studies, which allow assessment of implant biocompatibility to prevent the onset of inflammatory responses in the body [73,121,122]. Table 9 summarizes studies conducted on breast implants.

## 8. Summary

The use of breast implants undoubtedly contributes to increased patient confidence and self-esteem, as confirmed by numerous studies and survey results from individuals who have undergone the procedure [131,132,133]. Satisfaction rates related to improved body image range from 85% to 95% [131]. Patients’ psychological and sexual well-being, especially after mastectomy, is significantly improved, and implants additionally provide an aesthetically pleasing and natural breast appearance. However, it is important to note that, as with any introduction of a foreign material into the body, complications may occur [133].

The most common complication following breast implant placement is capsular contracture, which involves fibrosis and stiffening of the capsule surrounding the implant due to bacterial biofilm formation, potentially causing pain and deformities. Capsular contracture is observed significantly more often in patients who have undergone radiotherapy, as confirmed by studies involving women treated for breast cancer [134]. The literature indicates that the development of capsular contracture may be related to implant surface texture. Data showed that silicone implants exhibited a higher rate of capsular contracture: 7.2% in primary augmentation and 12.7% after reconstruction, compared with saline-filled implants [135].

Implant rupture represents another significant long-term complication, particularly occurring more frequently with silicone implants. Despite technological advances that have significantly reduced the risk of damage, it has not been completely eliminated. In the case of silicone implants, ruptures often occur asymptomatically. Leaked silicone may remain within the capsule or migrate to surrounding tissues and lymph nodes. According to current Food and Drug Administration (FDA) recommendations, routine screening in women with silicone breast implants should include magnetic resonance imaging three years after implantation, and every two years thereafter [27]. Regular monitoring is important, as the highest risk of asymptomatic implant rupture occurs 10 to 15 years after surgery and can lead to local migration of the material into surrounding tissues and, consequently, systemic infection. Following implant placement, allergic reactions, hematoma, and capsular contracture may also occur, potentially resulting in spontaneous implant displacement [69,136].

Ruptures of saline-filled implants are easy to detect, as the breast quickly loses volume due to fluid leakage. Saline is completely safe and is naturally absorbed by the body, making this type of implant one of the safest options [56].

Another rare but significant health risk is the occurrence of anaplastic large cell lymphoma (ALCL). This is a subtype of T-cell lymphoma that develops in the tissues surrounding the breast implant. It most commonly presents as breast swelling, which can occur from 2 up to 38 years after implantation (on average after approximately 8 years). It is currently believed that ALCL development is associated with textured implant surfaces. The FDA has reported a single case of BIA-ALCL in a patient with smooth breast implants. The occurrence of ALCL necessitates implant removal, and in more advanced cases, immunotherapy [137,138]. Other complications associated with breast implant placement include infections, postoperative hematomas, changes in nipple sensation, and deformities related to improper implant positioning [138]. The literature also describes the term Breast Implant Illness (BII), which encompasses a set of symptoms associated with breast implants, such as chronic fatigue, muscle and joint pain, memory and concentration disorders, headaches, and skin complaints. It is important to note that BII is not formally recognized as a medical condition. Diagnosis is primarily based on patient-reported symptoms and their presumed association with the implant [139]. Ultimately, the decision to undergo breast implant placement remains an individual choice, requiring careful consideration of all clinical aspects as well as long-term postoperative care.

## Figures and Tables

**Table 1:** Phenomena at the implant–tissue interface.

Implant–Tissue Interface Phenomenon	Description and Clinical Implications	References
Formation of a fibrous capsule around the implant	A natural process in which a layer of scar tissue forms around the implant	[41]
Excessive scarring and stiffening of the capsule around the implant—formation of capsular contracture	The fibrous capsule becomes thicker and stiffer as cells produce increased amounts of collagen fibers—this excess is problematic. The consequences include breast firmness, pain, deformity, and, in many cases, implant displacement.	[35]
Chronic presence of bacteria on the implant surface (biofilm)	A biofilm is a thin layer of bacteria on the implant surface, forming a structured microbial community adherent to that surface and encased in a self-produced extracellular matrix. Subclinical biofilms (most commonly composed of skin commensals such as *Staphylococcus epidermidis* and *Cutibacterium acnes*) have been identified on explanted devices, and their presence correlates with the severity of capsular contracture. A causal relationship has not been definitively established, and biofilm is currently regarded as one of several contributing factors within a multifactorial etiology.	[36,37]
Migration of silicone particles from the interior of the implant into the body	Even when the implant is not damaged, small amounts of material may come into contact with the body. This may lead to chronic inflammation and discomfort.	[40]

**Table 2:** Stages of physiological wound healing.

Stages of Wound Healing	Biological Mechanisms	References
Hemostasis	Platelet activation; fibrin clot formation; cytokine release, including TGF-β growth factors, leading to initiation of the inflammatory phase	[42,43,44]
Inflammation	Neutrophil and macrophage infiltration; release of proinflammatory cytokines (IL-1β, IL-6, TNF-α)	
Proliferative phase	Fibroblast proliferation; extracellular matrix (ECM) formation; granulation tissue formation	
Remodeling	ECM remodeling; replacement of type III collagen with type I collagen; scar formation	

**Table 3:** Stages of the foreign body response at the implant–tissue interface.

FBR Stage	Biological Mechanisms	References
Protein adsorption and provisional matrix formation	Implant surface coating with proteins: albumin and fibrinogen; formation of a provisional ECM dominated by fibrin	[34,46,47,48]
Acute inflammation	Neutrophil and macrophage infiltration; release of proinflammatory cytokines (IL-1β, IL-6, TNF-α)	
Chronic inflammation	Presence of numerous macrophages and lymphocytes; transition of macrophages from a proinflammatory (M1) to an anti-inflammatory (M2) phenotype	
Foreign-body giant cell (FBGC) formation	Fusion of macrophages into foreign-body giant cells (FBGCs) and their adhesion to the implant surface; extracellular matrix (ECM) formation	
Encapsulation	Increased deposition of type I collagen; formation of a fibrous capsule around the implant	

**Table 4:** Complications following breast implant placement.

Complications	Description and Clinical Implications	References
Implant damage	Implant damage may occur both during placement and after many years of use. Implant damage may lead to silicone gel leakage beyond the fibrous capsule into breast tissues or further, triggering a foreign body response with painful granulomas and extensive scarring. Studies indicate that women with extracapsular leakage are three times more likely to report a diagnosis of fibromyalgia.	[49]
Hematoma	It is a common early postoperative complication. It causes swelling, pain, and discomfort. Hematomas or bleeding are classified as clinical indications for subsequent surgical intervention. In some cases, hematoma evacuation requires temporary removal of the implant.	[50]
Infection	Infection of tissues surrounding the implant; in many cases, postoperative treatment is required.	
Implant displacement	Uncontrolled postoperative implant displacement, which may be associated with hematoma or excess fluid accumulation at the implant site. In many cases, reoperation and implant repositioning are required	[51]
Excessive stiffening of the capsule around the implant (capsular contracture)	The most common complication in patients. It results from excessive scarring, more often following hematoma, fluid accumulation, or infection	[50]
Lymph node enlargement due to the presence of the implant	Material (especially after rupture) may migrate to lymph nodes, causing their enlargement. A very dangerous condition requiring evaluation by a physician and often reoperation.	[49]

**Table 5:** Classification of Breast Implant Volumes and Their Impact on Cup Size, based on [61].

Size of Breast Implant	Volume	Approximate Cup Increase
Small	Approximately 100–250 cc	0.5 to 1
Medium	Approximately 250–400 cc	1 to 2
Large	Approximately 400–600 cc	2 to 2.5
Very large	Greater than 600 cc	2.5+

**Table 6:** Manufacturers’ catalogs of breast implants with characteristics of silicone gels.

Manufacturer	Material	Type	References
GC Aesthetics	Silicone gel: ParaGel^TM^	Highly cohesive, medical-grade gel enabling controlled distribution of the material within the implant, available in variants with different degrees of cohesiveness (Soft Cohesive and Natural Cohesive)	[71]
	SiloGel Twist™	Soft, stable, highly cohesive sixth-generation silicone gel	
	Silogel™	Sixth-generation, soft, stable, highly cohesive silicone gel that is highly resistant to rupture during implant use	[72]
POLYTECH	EasyFit Gel™	Softer gel—provides optimal adaptation of the implant to the chest	[73]
	Shapar Gel™	Firmer gel—stabilizes projection and may support the DualPlane technique as an internal bra	
Allergan Aesthetics	Natrelle INSPIRA^®^	The cohesive nature of the gel allows the production of implants of various shapes and provides a shape memory effect, characterized by a return to the original shape after exposure to external pressure.	[74]

**Table 7:** Types of breast implant surfaces [93].

Roughness	Surface Type
<50 μm	Macrotexture
10–50 μm	Microtexture
0–10 μm	Smooth

**Table 9:** Studies on breast implants.

Type of Study	Standard	Study Objective	Description of the Study	References
Mechanical testing of breast implants in the implantation-ready state	ISO 14607:2024 Non-active surgical implants—Mammary implants—Particular requirements [123]	The study aims to perform strength tests on silicone breast implants, including tensile and compression tests, to assess mechanical properties.	The implants consisted of shells made of medical-grade silicone rubber, approximately 0.5 mm thick, filled with high-viscosity medical-grade gel. For compression strength testing, two implants with a volume of 275 cm^3^ were selected. One implant was completely new, while the other underwent an aging process in saline at 37 °C for ten months. The test was conducted at a constant speed of 5 mm/min using a dynamometer; the internal pressure was estimated as the ratio of the applied load to the contact area between the implant and the compression plate. For tensile strength testing, sets consisting of fifteen samples aged at 37 °C, 60 °C, 75 °C, and 90 °C were used. Periodically, five samples from each set were collected for testing after 6 months, 1 year, and 2 years of aging. Tensile tests were performed at an extension rate of 50 mm/min. The new breast implant exhibited the highest load resistance, reaching a maximum force of approximately 7.650 kN at a final thickness of 8.44 mm. This corresponded to an internal pressure of approximately 2.35 bar. In the aged samples, a slight decrease in maximum force values was observed, suggesting gradual degradation of mechanical properties over time.	[124]
Surface topology tests of breast implants	-	The aim of the study was to use scanning electron microscopy (SEM) to evaluate implant surface roughness in order to investigate the influence of surface topography on cell adhesion and implant integration with surrounding tissues.	To assess the effect of SMI surface topography on bacterial biofilm formation, silicone patches were examined by SEM both in the initial state and after overnight inoculation with *S. epidermidis* or *S. aureus*. Untreated control SMI patches remained sterile. The external implant shell surface was also compared across different topographies (4 μm and 60 μm). Biofilm formation was confirmed on both textured surfaces, but it was markedly more complex on the Ra 60 μm surface. Individual cells and colonies were observed on the external surface of the smooth implant shell, whereas a dense biofilm was visible on the internal surface of the smooth shell, indicating a potential risk of biofilm formation in the event of implant rupture.	[65,125,126]
Surface roughness tests	-	The aim of this study was to assess the surface roughness of silicone breast implants.	The Ra parameter ranged from 0.2 ± 0.03 μm for smooth implants to 32 ± 7.0 μm for microtextured implants.	[91,127]
Surface wettability tests	-	The surface wettability study aims to determine whether the surface properties of silicone breast implants are hydrophilic or hydrophobic. This allows assessment of the implant potential for integration with surrounding tissues in the body.	Based on the conducted tests, the authors observed that implants made of PDMS (polydimethylsiloxane) exhibit a hydrophobic character, which affects biofilm formation and bacterial adhesion.	[127]
Tribological tests	-	The aim of this study was to assess the influence of silicone implant surface roughness on friction coefficient and the potential risk of inflammatory response.	Three types of implant surface were compared: a smooth surface (Ra = 2.7 ± 0.6 µm), which showed a mean friction coefficient of 0.46 ± 0.11; a microtextured surface (Ra = 32 ± 7.0 µm), with a friction coefficient of 1.20 ± 0.10; and a macrotextured surface (Ra = 80 ± 10 µm), which exhibited the highest friction (2.82 ± 0.15). The results showed that the smooth surface demonstrated the lowest friction coefficient and generated the smallest amount of wear debris	[128]
Statistical analysis	-	The aim of the study was to gather the experiences of consultant plastic surgeons performing cosmetic breast augmentation procedures in order to identify the most commonly used practices and preferences regarding surgical techniques.	The questions included, among other aspects, implant structure, volume, brand, and type of incision. A total of 75 consultant plastic surgeons responded. Textured breast implants were the most commonly used (82.7%), whereas smooth implants were more frequently chosen by surgeons performing a higher number of procedures annually (>50 cases/year). All respondents preferred inframammary incisions, while the most common implant placement was subglandular.	[129]
Applications to Clinical Practice	-	The aim of the study was to select the appropriate breast implant for patients based on their varying anatomical structures and body weight	The article presents case reports of three patients aged 31, 38, and 52. For each patient, implants of varying sizes were recommended, customized to preserve the natural shape and appearance of the breasts.	[130]

## Data Availability

No new data were created or analyzed in this study.
